# Control of ABA Signaling and Crosstalk with Other Hormones by the Selective Degradation of Pathway Components

**DOI:** 10.3390/ijms22094638

**Published:** 2021-04-28

**Authors:** Agnieszka Sirko, Anna Wawrzyńska, Jerzy Brzywczy, Marzena Sieńko

**Affiliations:** Laboratory of Plant Protein Homeostasis, Institute of Biochemistry and Biophysics, Polish Academy of Sciences, ul. Pawinskiego 5A, 02-106 Warsaw, Poland; jurek@ibb.waw.pl (J.B.); marsi@ibb.waw.pl (M.S.)

**Keywords:** abscisic acid, autophagy, brassinosteroids, cytokinins, hormone crosstalk, ubiquitin

## Abstract

A rapid and appropriate genetic and metabolic acclimation, which is crucial for plants’ survival in a changing environment, is maintained due to the coordinated action of plant hormones and cellular degradation mechanisms influencing proteostasis. The plant hormone abscisic acid (ABA) rapidly accumulates in plants in response to environmental stress and plays a pivotal role in the reaction to various stimuli. Increasing evidence demonstrates a significant role of autophagy in controlling ABA signaling. This field has been extensively investigated and new discoveries are constantly being provided. We present updated information on the components of the ABA signaling pathway, particularly on transcription factors modified by different E3 ligases. Then, we focus on the role of selective autophagy in ABA pathway control and review novel evidence on the involvement of autophagy in different parts of the ABA signaling pathway that are important for crosstalk with other hormones, particularly cytokinins and brassinosteroids.

## 1. Introduction

Plants are often subjected to various detrimental environmental stressors. Despite their sessile lifestyle, plants are able to maintain their internal equilibrium when exposed to external challenges. This balance, called homeostasis, can be achieved by internal mechanisms that alter cell metabolism, thanks to which the whole organism can maintain relatively constant conditions. Such rapid and appropriate genetic and metabolic acclimation, which is a crucial issue for plants’ survival, is maintained due to the coordinated action of plant hormones and cellular degradation mechanisms influencing proteostasis.

Protein homeostasis (or proteostasis) integrates cellular pathways that mediate biogenesis, folding, trafficking, and the degradation of polypeptides to maintain the required concentrations of all proteins that compose the proteome. This field has been intensively investigated by numerous research groups [1]. Proteins are often tagged for removal by ubiquitination. Polyubiquitinated proteins and protein aggregates are degraded via the ubiquitin–proteasome system (UPS) pathway and via the autophagy–vacuolar route. The UPS pathway is restricted in its ability to degrade aggregated proteins, which are too large to pass through the narrow proteasome entrance channel [2]. Thus, autophagy represents a major mechanism in plants for degrading macromolecular ubiquitinated protein aggregates as a response to stressors [3,4]. There is growing evidence that these two degradation systems are interconnected and form an integrated quality control network in response to general cellular stress [5]. Many reviews summarize the significance of ubiquitination in plant stress responses [6,7,8].

Phytohormones are not only involved in protein degradation mechanisms, but also play a pivotal role in plant responses to various stimuli. Although the relationships between them are poorly understood, it is generally accepted that extensive crosstalk and multiple regulatory loops are responsible for maintaining the balance of growth and stress responses [9].

Phytohormones are master regulators of plant growth, development, and stress response. Of them, abscisic acid (ABA) is rapidly accumulated in plants in response to abiotic stress [10]. Similarly to most other hormones, ABA is involved in developmental processes, including seed maturation, seed dormancy and germination, primary root growth, and flowering time control, and in the response to adverse environmental stressors [10,11]. Plants exposed to abiotic stress quickly activate the ABA signaling cascade, resulting in the activation of ABA-responsive transcription factors and the induction of ABA-responding genes. However, after achieving stress tolerance, it is necessary to terminate (or attenuate) the ABA pathway. The balance is maintained by the selective degradation of specific components of the ABA signaling pathway upon their tagging for decay by ubiquitination. Conventional degradation of the tagged components takes place via the proteasome; however, increasing evidence demonstrates a significant role of autophagy in controlling ABA signaling [9,12,13,14,15,16]. In addition to this interplay with degradation systems, an additional layer of complexity is provided by extensive crosstalk between ABA and other phytohormones [17,18,19,20].

The above issues have been frequently reviewed; however, although the field has been extensively investigated, new discoveries are constantly being provided. The purpose of this review is to update information on the selective degradation of components of the ABA signaling pathway, particularly on the control of ABA-responsive transcription factors and the role of selective autophagy in ABA pathway control. We also reviewed novel evidence on the involvement of autophagy in different parts of the ABA signaling pathway that are important for crosstalk with other hormones, particularly cytokinins and brassinosteroids.

## 2. Principles of ABA Perception and Signaling and Their Control by Selective Degradation Systems

**Figure 1:**
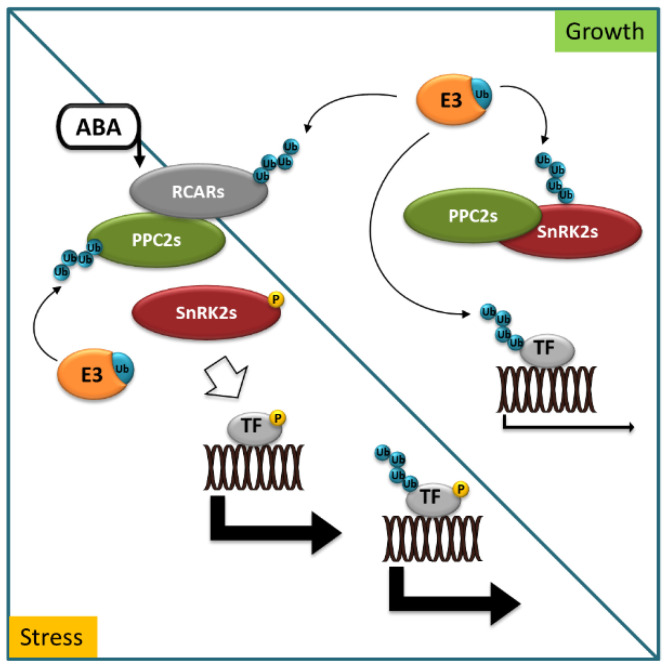
Abscisic acid (ABA) signaling in stress and growth. In growth-promoting conditions, ABA receptors (RCARs), protein kinases (SnRK2s) dephosphorylated by type 2C protein phosphatases (PP2Cs), and ABA-responsive transcription factors (TF) are marked for degradation with ubiquitin (Ub) by specific E3 ligases (E3). In stress conditions, ABA accumulates and binds to RCARs, allowing for interaction with PP2Cs to inhibit phosphatase activity. SnRK2s are released to phosphorylate and control the activity of downstream targets to trigger physiological responses. Blue circles indicate ubiquitination, yellow circles indicate phosphorylation events.

Reversible protein phosphorylation is a post-translational modification catalyzed by protein kinases and phosphatases. It acts as a molecular switch for almost all key events of cell metabolism and signaling in eukaryotes. Additionally, in the field of ABA signal transduction, protein kinases and phosphatases play pivotal roles, forming a reversible protein phosphorylation cascade, including ABA receptors (pyrabactin resistance/regulatory components of ABA receptor (PYR/RCAR)), type 2C protein phosphatases (PP2Cs), protein kinases (Snf1 (sucrose-non-fermentation 1)-related kinases subfamily 2, SnRK2s), and downstream targets. The key function of RCARs is to indirectly control the activity of SnRK2s, which phosphorylate numerous stress-activated targets in the response to ABA, including ABA-responsive transcription factors (TFs) [21,22]. This is accomplished through a negative regulatory pathway, involving RCARs, PP2C, and SnRK2 proteins and E3 ubiquitin ligases (Figure 1).

When ABA accumulates in cells in response to environmental stress or developmental cues, ABA binds to RCARs and triggers a conformational change that allows the binary complex (receptor–ABA) to physically interact with PP2Cs and inhibit its phosphatase activity. The various RCAR–PP2C heterodimers preclude binding of SnRK2s to PP2Cs, thus stimulating SnRK2s kinase activity. Consequently, SnRK2s are released to phosphorylate and control the activity of downstream factors to trigger physiological responses [23].

Several studies have suggested that plant responses to ABA are complemented by the enhanced degradation of PP2Cs by different E3 ligases (i.e., U-box type, RING type, and multimeric CRL type), which can facilitate ABA signaling and play an important role in preventing the excessive accumulation of PP2Cs. However, E3 ligases are not only able to enhance ABA signaling, but also facilitate the resetting of ABA signaling when tolerance to the stressor has been achieved [12].

## 3. Role of Ubiquitination and Selective Degradation in ABA Perception and Signaling

**Figure 2:**
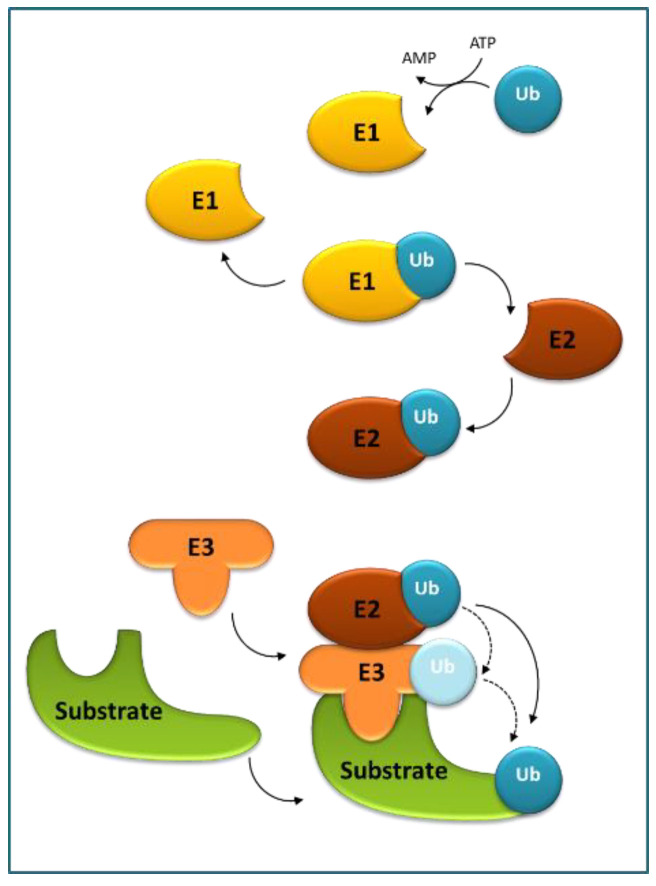
The ubiquitination cascade. Ubiquitin-activating enzyme (E1) activates ubiquitin (Ub) in an ATP-dependent manner, and next the thioester-linked Ub is transferred to Ub-conjugating enzyme (E2). Subsequently, E2 enzymes catalyze the attachment of Ub to a substrate using Ub ligase (E3). E3s, responsible for substrate recognition, transfer Ub either directly from E2 or form an E3–Ub intermediate prior to the transfer.

Ubiquitination is (next to phosphorylation) another reversible, post-translational modification, originally discovered as a critical element in highly regulated proteolysis and regarded as essential for many other cellular processes. Ubiquitin (Ub) serves as a reusable tag that decides the cellular fate of the proteins and is recognized by numerous Ub-binding proteins directing the target proteins for selective turnover [24,25,26]. Ub, often in the form of polymeric chains, is covalently attached to the target proteins through a three-step E1–E2–E3 conjugation cascade (Figure 2).

The process starts with E1 (or Ub-activating enzyme) in which Ub is activated in an ATP-dependent manner, through a thioester bond between the C-terminus of Ub and a cysteine residue of E1, and next the thioester-linked Ub is transferred onto a cysteine residue of E2 (or Ub-conjugating enzyme) by transesterification to form an unstable E2–Ub intermediate. Next, E2 enzymes catalyze the attachment of Ub to lysine (Lys) residues of target proteins using an E3 (or Ub ligase). E3 enzymes are responsible for substrate recognition and either directly transfer the Ub to the substrates from E2 or form an E3–Ub intermediate prior to the transfer. In successive rounds, additional Ub moieties can be attached to one of the seven lysine residues (Lys6, Lys11, Lys27, Lys29, Lys33, Lys48, and Lys63) or the N-terminal methionine (Met1) of Ub to produce poly-Ub chains. The end-product is a Ub–protein conjugate that contains an isopeptide bond between the C-terminal glycine residue of Ub and one of the Lys residues in the substrate. These structurally distinct ubiquitylation patterns are recognized by various effector proteins harboring a ubiquitin-binding domain (UBD) to result in diverse downstream signals. For example, poly-Ub chains are linked by Lys48 target substrates to the 26S proteasome for degradation, whereas poly-Ub chains are linked by Lys63 direct substrates to autophagosomes, both with the concomitant release of Ub moieties for reuse [27].

The E3 Ub ligases can be classified into single- and multi-subunit groups [28]. The single-subunit group includes several subfamilies based upon their mechanisms of action and the presence of specific domains: HECT (homology to E6–AP C terminus), RING (really interesting new gene), and U-box type E3s. The multi-subunit group, cullin-RING box1-ligases (CRLs), are further divided into four subfamilies: SCF (S phase kinase-associated protein 1–cullin 1–F-box), BTB (Bric-a-brac–Tramtrack–Broad complex), DDB (DNA damage-binding domain-containing), and APC (anaphase-promoting complex).

Three E2 enzymes, UBC32, UBC33, and UBC34 play a negative role in drought stress response and ABA signaling [29], and numerous E3 ligases are involved in the inhibition of ABA signaling in optimal conditions, ABA induction upon environmental stress, and ABA attenuation after achieving stress tolerance [12]. The currently known components of ABA perception and signaling and E3 ligases involved in the process are listed in Table 1.

### 3.1. E3 in ABA Perception and ABA Signaling Cascade

**Figure 3:**
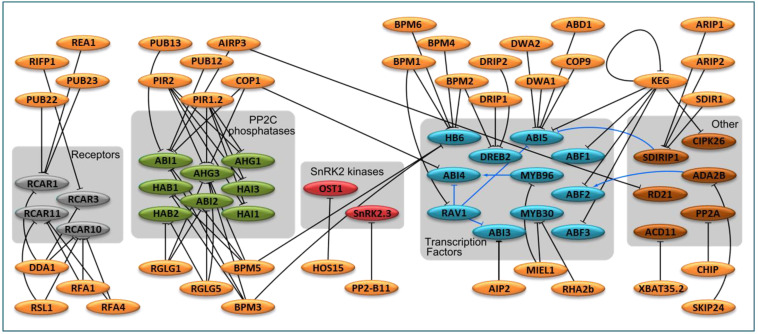
The network of E3 ligases and their targets involved in ABA signaling. E3 ligases are shown in orange, and their targets in ABA signaling pathway are colored according to target protein function (receptors in gray, PP2C phosphatases in green, transcription factors in blue, and other related components in terracotta). Different groups of target proteins are also shaded and labeled. Activation is shown by arrows, and inhibition by T-shaped bars. Black lines show interactions between proteins, while blue lines denote transcriptional control. The references are provided in Table 1.

The E3 ligases involved in controlling the ABA signaling pathway, as well as their targets (Table 1), are linked together, creating a large network of interactions (Figure 3). When dividing this network according to a functional group of E3 targets, it can be observed that the majority of E3s are highly selective with respect to their targets and are usually limited to a single functional group of the ABA signaling pathway. This is particularly evident in the case of SnRK2s, where only two ‘E3-target’ pairs have been identified thus far, namely, HOS15 ubiquitinates SnRK2.6/OST1, while PP2-B11 ubiquitinates SnRK2.3. It is not clear whether so few Ub ligases controlling SnRK2s results from the paucity of experimental data, or if this signaling step is weakly controlled at the level of protein ubiquitination.

At least eight E3 ligases were found to target at least four ABA receptors (Figure 3). Although some of them have two or three known targets, they exclusively target ABA receptors and no other element of the ABA signaling pathway.

The interaction network becomes slightly more complex for the eight PP2Cs, having known interactions with 10 E3s (Figure 3). Although all eight PP2Cs are targeted by more than one Ub ligase, and most of these E3s target more than one protein in the ABA signaling pathway, only four of these E3s interact with proteins other than PP2Cs. Three E3 ligases are common for some PP2Cs and two transcription factors involved in the ABA response.

Thus far, 11 transcription factors, involved in regulation by ABA, were found to be targeted by 17 E3 ligases (Figure 3). Among them, 13 are specific to transcription factors and they usually target only one or two proteins. Transcription factor HB6 is under the control of six BPM Ub ligases (BPM1-6). Transcription factor ABI5 is directly controlled by ubiquitination by five Ub ligases (DWA1, DWA2, ABD1, COP9, and KEG). ABI5 is also controlled at the transcriptional level by RAV1 and SDIRIP1, which are controlled by ubiquitination. The Ub ligase KEG controls four transcription factors (ABI5, ABF1, ABF2, and ABF3) and kinase CIPK26; thus, KEG appears to be one of the most important ligases controlling the ABA signaling pathway.

### 3.2. E3 in ABA-Responsive Transcription Factors

Ub-mediated protein degradation can affect the gene expression needed to mitigate the potential negative effects of environmental stress by modulating the abundance of transcription factors. Additionally, under non-stress conditions, E3 ligases may prevent transcription by targeting the transcription factor for degradation. Transcriptional changes of ABA-responsive genes are mediated by a number of transcription factors, including members of the basic leucine zipper (bZIP), AP2/ERF, R2R3, and B3 families [10]. The abundance of many of these transcription factors is strictly guarded by numerous E3 ligases (Figure 3, Table 1).

#### 3.2.1. E3 Directly Binding ABA-Responsive Transcription Factors

A RING-type E3 ligase, ABI3-INTERACTING PROTEIN 2 (AIP2), was the first E3 ligase to be implicated in the regulation of ABA signaling because it mediates ABSCISIC ACID INSENSITIVE 3 (ABI3) ubiquitination and its destruction through the 26S proteasome [48,49]. The abundance of AIP2 transcripts increases in response to ABA and corresponds well with the decrease in ABI3 protein levels. The aip2 mutant was found to have higher levels of ABI3 and be hypersensitive to ABA. The ortholog of AIP2, OsDSG1 (DELAYED SEED GERMINATION 1), has been described in rice [71]. It also physically interacts with OsABI3 and has E3 Ub ligase activity, while negatively regulating tolerance to salt and drought stress.

Although ABI4 was initially demonstrated to act as a positive regulator in the ABA signaling cascade, several studies have revealed that it is involved in the control of a variety of other cascades and crosstalk among several phytohormones [72]. ABI4 either stimulates or represses the transcription of its target genes, which is consistent with its diverse biological functions. Due to the ABI4 protein’s instability [73], the E3 ligases that are directly responsible for its degradation have remained elusive thus far. ABI4, together with ELONGATED HYPOCOTYL 5 (HY5), have been described to form an antagonistic module that controls seedling and chloroplast development during the transition from dark to light [49]. ABI4 promotes and HY5 inhibits the expression of CONSTITUTIVE PHOTOMORPHOGENIC 1 (COP1), the main repressor of photomorphogenesis. In turn, to ensure proper crosstalk between ABI4 and HY5 during the seedling greening process, COP1 targets ABI4 and HY5 for degradation in light and dark conditions, respectively. COP1 promotes ABI4 degradation, as evidenced by in vitro pull-down and in vivo co-immunoprecipitation assays [50].

The well-studied ABI5 is a nucleo-cytoplasmic bZIP-type transcription factor that acts downstream of ABI3. A monomeric RING-type E3 and two CUL4-RING E3 ligase (CRL) complexes have been implicated in controlling ABI5 stability and attenuating ABA signaling. CRL complexes, which are the largest class of Ub-modification enzymes in Arabidopsis, consist of a scaffold protein, one cullin protein, and an adaptor protein responsible for substrate recruitment [74]. DWD HYPERSENSITIVE TO ABA1 and ABA2 (DWA1 and DWA2, respectively) and ABA-HYPERSENSITIVE DCAF 1 (ABD1), the substrate receptors in CRLs, are able to recognize ABI5 in the nucleus to negatively regulate its stability via ubiquitination and subsequent proteasomal degradation [44,46]. Both dwa1dwa2 double mutants and abd1 mutants displayed ABA-hypersensitive phenotypes during seed germination and seedling growth and exhibited an accumulation of ABI5 following exposure to the hormone [46]. ABI5 did not accumulate in the dwa1dwa2 mutant in the absence of ABA, which agrees with CRL being required to regulate transcription factor abundance in the presence of the hormone. Importantly, in the absence of ABD1, proteasome-dependent degradation of ABI5 after the removal of ABA is slower, implying that the E3 ligase is involved in the termination of ABA responses [46]. ABI5 is also targeted by Ub-dependent proteolysis by a RING-type E3 ligase KEEP ON GOING (KEG) [45]. There is a feedback loop between ABA signaling, KEG, and ABI5, in which KEG promotes ABI5 degradation to suppress ABA signaling, while ABA accelerates KEG self-ubiquitination and degradation, resulting in ABI5 accumulation and ABA response promotion. KEG is localized to the trans-Golgi network/early endosome; therefore, it is believed that KEG marks ABI5 for degradation before it reaches the nucleus and activates ABA responses [52]. Additionally, another element that regulates ABI5 protein turnover is ABI FIVE BINDING PROTEIN (AFP) [75]. AFP belongs to a small family of proteins, AFP1–4, that can interact with ABI5. Although AFP is not an E3 ligase per se, it facilitates ABI5 proteasomal degradation in nuclear bodies, however the precise mechanism is not known. In rice, the AFP orthologue MEDIATOR OF OsBZIP46 DEACTIVATION AND DEGRADATION (MODD) interacts with the ABI5 orthologue OsbZIP46 to promote its ubiquitination by the E3 ligase OsPUB70, stopping ABA signaling [76]. Recently, another layer of regulation was discovered for ABI5 stability. The COP9 signalosome (CSN) regulates seed germination by facilitating ABI5 degradation [47]. CSN is a multi-subunit protein complex that may either inhibit or promote CRL complex activity. The CSN5A subunit was shown to interact with ABI5; however, the precise mechanism by which CSN5A regulates ABI5 stability will be a challenge for future studies. Finally, post-translational modifications of ABI5, such as phosphorylation and SUMOylation, may also play a role in ABI5 proteasomal turnover [75,77,78].

In addition to ABI5, KEG also targets two other bZIP transcription factors, ABRE-BINDING FACTORS (ABF) 1 and ABF3, for degradation [51]. ABF1 and ABF3, which are also involved in seed germination and stress responses, are stabilized by ABA. Other ABFs, including ABF2 and ABF4, may also be involved in the KEG-mediated degradation pathway. The phenotype of KEG mutants is quite severe compared to other ABA mutants, and growth arrest occurs soon after germination in the absence of the hormone [68]. This lethality of the KEG mutation might be explained by the fact that KEG targets multiple components (CIPK26, ABI5, and ABF1/3) of the ABA signaling pathway. The accumulation of its substrates may lead to the misregulation and activation of other pathways independent of ABA [68].

DEHYDRATION-RESPONSIVE ELEMENT-BINDING PROTEIN 2A (DREB2A) is a key transcription factor controlling the expression of numerous genes responsive to drought, heat, and salt [79,80]. DREB2A only accumulates in transgenics capable of attaching Ub molecules to DREB2A in non-stress conditions within the nucleus [54]. DREB2A is stable in drip1drip2 double mutants, which present significantly higher survival rates in response to dehydration [54,81]. The situation is different under stress conditions, where the nuclear-localized A20/AN1-type zinc finger protein, STRESS ASSOCIATED PROTEIN 5 (SAP5), functions as an E3 ligase that promotes the degradation of DRIP1/2 to release and stabilize DREB2A [82]. Notably, SAP5 shows a preference for adding linear and Lys63-linked chains compared to Lys48-linked chains [83]. This emphasizes the potential role of SAP5 in the selected trafficking of polyubiquitinated proteins to other 26S proteasome degradation systems, such as autophagy. DREB2A abundance increases upon exposure to heat stress. However, to attenuate the stress response, the level of the transcription factor gradually decreases over time due to the interaction with a MATH DOMAIN-CONTAINING BTB PROTEIN (BPM), which functions as a DREB2A receptor in a Cul3 BPM1-6 RING E3 ligase complex [55]. DREB2A contains a 30-amino acid serine threonine-rich region, referred to as the NEGATIVE REGULATORY DOMAIN (NRD), which is necessary for Cul3 BPM1-6 RING-depended degradation. Biochemical data proved that the NRD domain, by itself, is capable of adequately interacting with BPM proteins. Deletion of the NRD increases DREB2A stability and carries on its constitutive action in improving tolerance to drought and heat stress [79]. To add further complexity, a recent study discovered that SUMO has a positive regulatory role in the action of DREB2A. The SUMOylation of Lys163, an adjacent lysine to the NRD, interferes with the BPM–DREB2A interaction, stabilizing DREB2A [84]. This SUMOylation process is induced by heat shock, thus tuning the degradation rate of DREB2A in response to abiotic stress.

The same Cul3–BPM1-6 RING E3 complex is also engaged in the degradation of yet another transcription factor of ABA signaling, HOMEOBOX-LEUCINE ZIPPER (HD-ZIP) ATHB6 [56]. In contrast to DREB2A, ATHB6 is a negative regulator of ABA responses, including drought and heat tolerance [79,80,85]. BPM silencing, as well as ATHB6 overexpression, leads to an increase in the level of the ATHB6 protein together with the decrease in its ubiquitination status. However, overexpression of BPM6 accelerated the turnover of ATHB6, indicating that BPMs influence ATHB6 stability [56]. Furthermore, BPM silencing results in ABA insensitivity and reduced stomatal closure, leading to increased transpiration and water loss, indicating that BPMs play a positive role in ABA signaling [56,86]. Under non-stress conditions, BPMs promote the proteasome-dependent degradation of ATHB6. This turnover of ATHB6 can be inhibited by ABA and may serve to attenuate ABA responses. Furthermore, all six BPM proteins can interact with three different HD-ZIP transcription factors (ATHB5, ATHB6, and ATHB16), implying that this family of E3 ligases may regulate other processes as well [56]. Subsequently, it was reported that BPM1 can interact with another transcription factor, RAV1 (related to ABI3/VP1), which negatively regulates the transcription of ABI3/4/5, unless it is phosphorylated by SnRK2s [60,61].

The RING-type E3 ligase MYB30-INTERACTING E3 LIGASE 1 (MIEL1) was first described to control the abundance of R2R3-type transcription factor MYB30 that negatively regulates ABA signaling and activates defense and HR responses [59,87]. MIEL1 expression is rapidly downregulated after bacterial infection to allow MYB30 accumulation, thus promoting defense and the hypersensitive response (HR), and the miel1 mutant displays enhanced resistance responses after inoculation with bacteria [59]. In addition to its role in the control of biotic stress, MIEL1 regulates abiotic stress in an ABA-dependent manner by targeting the transcription factor MYB96, which normally promotes the expression of ABI4 [57]. In the absence of ABA, MIEL1 accumulates and promotes the degradation of MYB96 to silence ABA signal transduction, whereas in the presence of the hormone, MIEL1 is degraded by UPS, promoting MYB96 transcriptional activity. According to these findings, MIEL1 may facilitate the ABA-mediated interplay between biotic and abiotic stress [88]. As an added complication, in addition to MIEL1, another RING E3 ligase, RING-H2 FINGER PROTEIN 2B (RHA2B), is also able to bind and promote the degradation of MYB30 in an ABA-dependent manner during drought response [58].

#### 3.2.2. E3 Indirectly Influencing the Activity of ABA-Responsive Transcription Factors

In contrast to the E3 ligases described above and defined as negative regulators of ABA signaling, E3 ligase SALT- AND DROUGHT-INDUCED RING FINGER 1 (SDIR1) is genetically characterized as a positive regulator. The sdir1 mutant has reduced sensitivity to ABA in seed germination and early seedling growth assays and reduced stomatal closure in response to ABA, while overexpression of SDIR1 leads to quite opposite phenotypes [65,89]. Furthermore, rice OsSDIR1 is also a functional E3 ligase, and its overexpression leads to markedly increased drought tolerance [90]. SDIR1 interacts with its substrate SDIR1-INTERACTING PROTEIN 1 (SDIRIP1) at the endoplasmic reticulum membrane to promote its degradation via the 26S proteasome. SDIRIP1 negatively regulates ABI5 expression to impact ABA-mediated seed germination and responses to salt [65]. SDIRIP1 RNAi lines are ABA hypersensitive, suggesting that SDIRIP1 functions as a negative regulator of ABA signaling. Another RING-type E3 ligase has been described to target SDIRIP1 in the cytosol. AtAIRP2 regulated the turnover of SDIRIP1 in cell-free degradation and protoplast co-transfection assays [64]. AtAIRP2 and SDIR1 have combinatory roles in seed germination, as evidenced by reciprocal complementation of the ABA- and salt-insensitive germination phenotypes of sdir1 and atairp2 mutants, respectively. Recently, another link that could promote SDIRIP1 degradation has been described [91]. RESPONSE TO LOW SULFUR 1–4 (LSU1–4), proteins, belonging to a small plant-specific protein family, were identified as important stress-related hubs that bind to SDIRIP1 [91]. LSUs are also able to bind to NBR1, a selective autophagy receptor; therefore, it is tempting to speculate that LSUs might serve as a bridge to facilitate the autophagic degradation of SDIRIP1.

In Arabidopsis, ABA plays a major role in the drought stress response with a well-described cis-acting ABA-RESPONSIVE ELEMENT (ABRE) involved in transcriptional activation [92]. Under drought stress conditions, transcription factor ABF2 binds to the ABRE sequence of drought-responsive genes. ABF2 recruits the ADA2B–GCN5 protein complex, in which ADA2B is a transcriptional co-activator protein, while GCN5 acts as a histone acetyltransferase [93,94]. A recent study revealed that ADA2B is targeted for proteasomal degradation by the At1g08710 protein [62]. That F-box protein is localized in the nucleus and forms an SCF complex by interacting with SKP1 and cullin1 proteins. At1g08710 mutant plants present a better survival rate under water deficit conditions and a high accumulation of the ADA2B transcript following ABA treatment. Additionally, transcripts of drought-responsive genes RD22, RD29A, and ABI3 were upregulated under drought stress. These results suggest that F-box protein At1g08710 imparts drought stress tolerance through ADA2B degradation. This very recent report proves that ABF2 is an in vitro ubiquitination substrate of KEG [53].

#### 3.2.3. Links to UPS and Further Layers of Complexity

UPS selectivity is accomplished not only at the ubiquitination level, but also by the proteasome itself [95]. The 19S regulatory particle (RP) of the 26S proteasome confers ATP dependence, as well as substrate specificity to the holoenzyme, especially for substrates bearing a poly-Ub tag. One of the subunits of the RP in Arabidopsis is RPN10. It is essential for the degradation of ABI5 by the proteasome, as evidenced by the increased ABA sensitivity and ABI5 accumulation in the rpn10-1 mutant. This suggests that ABI5 turnover is accomplished through the interaction between ABI5 and RPN10. It is not known whether this interaction is direct or indirect, dependent or independent of the ubiquitination status of ABI5, but it promotes its association with proteasomal proteases. This association may be prevented by the ABA signal which stabilizes ABI5 [96].

The regulation of the stability of ABA-related transcription factors is complex and multilayered. A single E3 ligase such as KEG or BPM can target multiple proteins. Alternatively, a single stress-related protein can be targeted for degradation by multiple E3 ligases. As described, ABI5 abundance is regulated by at least five different E3 ligases, which are used to inhibit its activity under different circumstances [97]. Such a mechanism may rapidly integrate different inputs to control the level of a key transcription factor, leading to the appropriate response. Furthermore, a single Ub ligase can conjugate Ub to substrates in a variety of ways (e.g., Lys63- vs. Lys48-linked Ub chains), depending on the signal obtained or the identity of the substrate, leading to different outcomes. An interesting example of such regulation is rice RING E3 ligase IPA1 INTERACTING PROTEIN 1 (IPI1), which ubiquitinates IDEAL PLANT ARCHITECTURE 1 (IPA1) which is involved in plant development [98]. By adding Lys48-linked poly-Ub chains, IPI1 promotes the degradation of IPA1 in panicles, while it stabilizes IPA1 in shoot apexes by adding Lys63-linked poly-Ub chains.

### 3.3. Other E3 Ligases Known to Modify ABA-Mediated Responses

Numerous E3 ligases have been linked to ABA-regulated responses; however, in many cases, their targets have not yet been characterized. These enzymes have been categorized based on their functions as positive and negative modulators of ABA signaling (Table 2 and references therein). In many cases, it is also unclear whether they are present in the same growth conditions, cell types, organs, or developmental stages.

## 4. Autophagy as a Cellular Degradation Pathway and a Part of Intracellular Trafficking

A multistep ubiquitination process resulting in the attachment of Lys48- and Lys63-linked poly-Ub tags is used to label proteins degraded by the 26S proteasome (a part of UPS) and via autophagy, respectively. UPS is the key regulator of proteostasis; however, the proteasome and autophagy complement each other in the maintenance of appropriate protein balance in plant development and stress response [5]. Several types of autophagy exist; however, macroautophagy is better described, and it will be called autophagy hereafter. It is characterized by the presence of autophagosomes, double-membrane vesicles surrounding cargos designed for degradation and delivering it to the vacuole, after fusion of the external autophagosomal membrane with the tonoplast [3,128]. The selectivity of autophagy is achieved due to the involvement of selective autophagy cargo receptors. They recognize cargo and dock to the autophagosomes [129,130]. Autophagy, in contrast to the 26S proteasome, can not only degrade proteins and protein aggregates, but also entire organelles or their parts. Therefore, it must be well controlled to avoid excessive degradation of the cellular content. It is negatively regulated by the Target of Rapamycin (TOR) kinase, which, along with its effectors, is active under nutrient-rich conditions when it upregulates cell growth and translation, but it is inactive during nutrient deficiency. The TOR kinase complex is modulated by diverse upstream inputs to phosphorylate different proteins in the nucleus, nucleolus, and cytosol, thus controlling transcription, the cell cycle, rRNA transcription, ribosome biogenesis, translation, and metabolism, which are pivotal to cell proliferation and growth [131,132].

Autophagy is a part of the intracellular vesicular system; therefore, extensive interlinking exists between autophagy and trafficking pathways, and functional endocytic and exocytic pathways are essential for efficient autophagy flux in all eukaryotes [27,133,134,135]. Autophagy controls degradation, and exocytosis (secretion) is responsible for the transport of cargo to the plasma membrane or extracellular space, while endocytosis transports cargo from the plasma membrane or extracellular space to intracellular organelles [136,137,138]. Plasma membrane receptors and transporters must be tightly regulated, and part of this control is mediated by their ubiquitination and recruitment to endosomal sorting complexes (ESCORTs) by proteins interacting with ESCORTs, of which, some are capable of Ub-binding [138,139]. Actin and microtubule cytoskeletons and their reorganization play an important role in the control of all three processes [140,141].

## 5. Role of TOR Kinase, Autophagy, and Intracellular Trafficking in the Control of ABA Signaling

Various elements of ABA signaling are ubiquitinated and degraded via the 26S proteasome [6,7,100]. The RPN10 subunit of the 26S proteasome controls ABA signaling, as demonstrated by rpn10-1 mutants that were more sensitive to ABA [96]. Interestingly, the same research group determined that RPN10 functions as a selective cargo receptor in proteaphagy (autophagic degradation of 26S proteasome) [142]. Data on the interplay of ABA with autophagy are still limited, but growing evidence indicates that they can co-regulate each other. For example, ABA activates autophagy by reducing the ATG4 persulfidation level. The enzymatic activity of ATG4 is reversibly regulated by sulfide, and this regulation effectively controls autophagy flux. ATG4 is necessary for processing newly synthesized ATG8 proteins that can be further lipidated to progress autophagy [143]. However, some data suggest that elevated autophagy might induce ABA responses. For example, the overexpression of banana ATG8f modulated drought stress resistance in Arabidopsis [144], and the overexpression of NBR1 in Arabidopsis resulted in increased ABA signaling [145].

**Figure 4:**
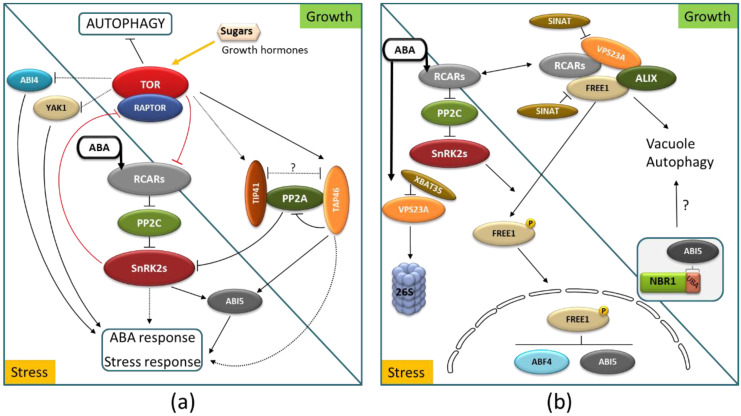
Links of autophagy with ABA signaling in growth and stress responses. (**a**) Reciprocal regulations of TOR and ABA signaling pathways. Red lines mark the main regulation circuit, while the other TOR–ABA connections are marked by the black lines. (**b**) Links of ABA with elements of vesicular transport and an autophagy cargo receptor NBR1. For explanations and references, see the text.

TOR kinase might be involved in the synthesis and/or distribution of ABA [146,147]. Additionally, the reciprocal regulation of ABA signaling and TOR kinase has been reported [148]. In growth-promoting conditions, the phosphorylation of ABA receptors by TOR disrupts their interaction with PP2C phosphatases, which can now interact with SnRK2s. This leads to the inactivation of SnRK2 kinases, thus keeping the ABA response silent. Under stress, ABA-activated SnRK2s induce the ABA response but also inactivate TOR and, in turn, activate autophagy [148]. There are additional indications for TOR–ABA connections. For instance, protein phosphatase 2A (PP2A)-associated proteins TAP46 and TIP41 indirectly influence ABA signaling, while they are also involved in TOR signaling [149,150,151,152]. Secondly, transcription factor ABI4, a key player integrating ABA responses, participates in TOR signaling during seed to seedling transition stages [72,153]. Moreover, YET ANOTHER KINASE 1 (YAK1), which was reported to participate in ABA responses, is directly or indirectly repressed by TOR to promote maximal meristem activity and size [154,155]. The molecular details of some of these links remain to be characterized. The known elements of ABA–TOR crosstalk are shown in Figure 4A.

Furthermore, there is an interplay of ABA signaling with endocytosis, exocytosis, the cytoskeleton, and selective targeting of ABA-responsive transcription factors (Figure 4B). The activity of soluble and membrane-located ABA receptors is regulated by proteins linked to endocytosis and autophagy. The connection of some RCARs with the proteins interacting with ESCORT complexes, such as VACUOLAR PROTEIN SORTING23A (VPS23A) [156], FYVE domain protein required for endosomal sorting 1 (FREE1) [157,158], or ALG-2 INTERACTING PROTEIN-X (ALIX) [159], have been revealed. The turnover of ABA receptors is controlled by the targeted ubiquitination of their partners. The XBAT35 E3 ligase is a positive regulator of ABA responses because it accelerates the turnover of VPS23A through the 26S proteasome, thereby reducing vacuolar degradation of RCAR10/PYL4 [160]. In the absence of ABA, the ubiquitinated RCAR10/PYL4 and RCAR11/PYR1 are recognized by VPS23A and FREE1 and targeted for vacuolar degradation. In the presence of ABA, VPS23A and FREE1 are targeted by the SINAT E3 ligase and directed to proteasomal and vacuolar degradation [161]. The termination of ABA responses leads to autophagic clearance of the excess of SINAT–VPS23A and SINAT–FREE1 complexes and the reset of cellular homeostasis.

ABA-mediated stomatal movement is also regulated by the exocyst subunit Exo70B1 [162]. Moreover, ABA mediates microtubule disorganization, which can influence all types of vesicular trafficking [109,110]. The links via Rho GTPases/Rho of Plants (ROP), which not only regulate numerous cytoskeleton and vesicular trafficking-based processes but also suppress ABA-induced responses, have also been revealed [163,164,165].

Interestingly, ABA also induces Arabidopsis multi-stress regulator tryptophan-rich sensory protein-related (TSPO), which acts as a heme scavenger and binds the excessive or deleterious heme and then is targeted for degradation through autophagy [163,166].

The ABA-autophagy links also take place at the level of ABA-responsive transcription factors. Recently, an interesting possibility for the regulatory circuit between selective autophagy and ABA-responsive transcription factors was suggested by the observation that ABI3, ABI4, and ABI5 interact with selective autophagy cargo receptor NBR1 [145]. Moreover, ABA-activated SnRK2s phosphorylate FREE1, allowing for its nuclear import. In the nucleus, FREE1 interacts with the basic leucine zipper transcription factors ABA-RESPONSIVE ELEMENTS BINDING FACTOR4 (ABF4) and ABA-INSENSITIVE5 (ABI5) to reduce binding to the cis-regulatory sequences of downstream genes and diminish ABA responses [167].

Both ABA signaling and autophagy can also be involved in crosstalk related to ROS (reactive oxygen species) homeostasis. For example, autophagy affects stomatal opening by controlling ROS production in guard cells [168] and regulates glucose-mediated root meristem activity by affecting ROS homeostasis [169]. Conversely, ABI5 regulates ROS during seedling germination by CAT1 catalase activation [170], and ABI4 (acting antagonistically to ETHYLENE-INSENSITIVE3 (EIN3)) regulates ROS accumulation by modulating ascorbic acid biosynthesis [171]. TOR also controls the ROS level by regulating mitochondrial activity [172].

## 6. Possible Involvement of Autophagy in ABA Crosstalk with Other Hormones

ABA is involved in extensive crosstalk with other phytohormones [19,72]. Up to now, autophagy has been suggested to participate in only two of such crosstalk events, ABA–cytokinins (CKs) and ABA–brassinosteroids (BRs). It is worth noting that CKs and BRs, similarly to many other hormones, regulate various aspects of plant growth antagonistically to ABA.

**Figure 5:**
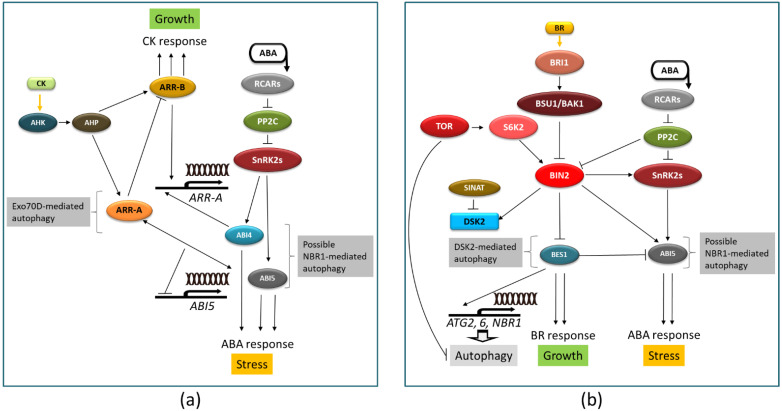
Interplay of autophagy with ABA–cytokinin crosstalk (**a**) and ABA–brassinosteroid crosstalk (**b**). Yellow arrows mark CK and BR signals. Elements of the CK signaling pathway (AHK, histidine kinase receptor(s); AHP, histidine phosphotransfer protein(s)) and BR signaling pathway (BRI1, leucine-rich repeat receptor-like kinase family; BSU1/BAK1, protein kinases; BIN2, GSK3-like kinase; BES1, transcription factor) are marked in panels A and B, respectively. See text for further details.

ABA–CK crosstalk is particularly apparent in the regulatory loop consisting of type-A and -B response regulators (ARRs) and SnRK2s [173]. Unlike type-B ARRs, which lack a DNA binding domain, type-A ARRs act as negative regulators of cytokinin signaling. Phosphorylation in response to cytokinin stabilizes a subset of the type-A ARRs. Plants modulate responses to CKs, at least in part via the autophagic regulation of type-A ARRs mediated by members of the EXO70D, a subclade of the EXO70 gene family, which act as receptors to recruit type-A ARRs to the autophagosome for subsequent degradation [174]. Additionally, type-A ARRs interact with ABA-responsive transcription factor ABI5 and downregulate ABI5 transcription [175]. The other ABA-responsive transcription factor ABI4 activates ABI4 transcription. Moreover, the interaction of ABI4 and ABI5 with selective autophagy cargo receptor NBR1 with ABI4 and ABI5 suggest that both transcription factors might be degraded via autophagy in an NBR1-dependent manner [145]. A fragment of the CK signaling pathway with ABA–CKs-autophagy interplay is illustrated in Figure 5A.

BR signaling has recently attracted attention [17,176,177,178,179]. The cross-regulation of ABA- and BR-signaling pathways is marked in Figure 5B. BRASSINOSTEROID-INSENSITIVE 2 (BIN2) interacts with central components of the ABA signaling pathway, such as bZIP transcription factor ABI5 [180] and protein phosphatase 2C ABI1 [181], and phosphorylates SNF1-related protein kinases SnRK2.2 and SnRK2.3 [182]. Moreover, ABA promotes BR signaling via ABI1/2, which, upon binding to BIN2, leads to the reduced phosphorylation of BRI1-EMS-SUPPRESSOR 1 (BES1) [183]. The links to autophagy are apparent on several levels. TOR, a negative regulator of autophagy, positively regulates BR signaling via RPS6 kinase 2 (S6K2), which, in turn, phosphorylates BIN2 [184]. In addition, transcription factor BES1, an important element of BR signaling, is selectively degraded via autophagy after being targeted to autophagosomes by selective autophagy cargo receptor DSK2 [185]. Interesting crosslinks have been observed in tomato, where autophagy-related genes ATG2, ATG6, and NBR1 are induced by BES1, which results in the induction of the autophagy pathway under chilling stress [186].

## 7. Closing Remarks

Perception and signaling of stress-triggering conditions are crucial for plant growth and development. Similarly, the control of ending these signaling events when they are no longer needed and reestablishing homeostasis is important. Ubiquitination, precisely provided by dedicated E3 ligases, is a universal mark for protein degradation. This mark is recognized by two major proteolytic pathways: UPS and autophagy. There are many unanswered questions on how plants coordinate and choose between these different proteolysis pathways to target the same or similar regulatory components. The clue might be in poly-Ub marks that have different architecture. However, what determines how particular E3 ligases structure the Ub chain is unknown.

During plant growth and development, as well as under stress, different phytohormones are mutually antagonistic and synergistic. Those phytohormones regulate autophagy, and vice versa, autophagy impacts the balance of phytohormones. It was recently discovered that autophagy is involved in the crosstalk of ABA with two hormones, CKs and BRs. However, other hormones are also regulated by ubiquitination and autophagy involvement in the crosstalk of ABA with the other hormones is quite plausible. Although the field is actively investigated, there is still a lot to discover. The future challenge is to identify phytohormone-related proteins that are specific targets of autophagy that are in line with their receptors. This knowledge would extend our understanding about stress regulation and, consequently, provide some new approaches for crop improvement, eventually leading to an increase in stress tolerance and yield.

## Figures and Tables

**Table 1:** ABA pathway components targeted by E3 ligases.

Pathway Component	Specific Target	E3 Ligase [References] (Remarks)
ABA Receptors	RCAR1/PYL9	DDA1 [30]; REA1 [31]; PUB22 [32]; PUB23 [32]
	RCAR3/PYL8	RIFP1 [33]; DDA1 [30]
	RCAR10/PYL4	DDA1 [30]; RSL1 [34]; UBC26 (E2)-RFA4 (E3) [35]; RFA1 [35]
	RCAR11/PYR1	RSL1 [34]; RFA1 [35]; RFA4 [35]
Type 2C protein phosphatases (PP2C)	ABI1	PUB12 [36]; PUB13 [36]; BPM3 [37]; BPM5 [37]; UBC27/AIRP3 [38]; COP1 [39]
	ABI2	RGLG1 [40]; RGLG5 [40]
	HAB1	BPM3 [37]; BPM5 [37]
	HAB2/NHL29	RGLG1 [40]; RGLG5 [40]
	AHG1	PIR1.2 [41]; PIR2 [41]
	AHG3/PP2CA	RGLG1 [40]; RGLG5 [40]; PIR1.2 [41]; PIR2 [41]; COP1 [39]; BPM3 [37]; BPM5 [37]
	HAI1/SAG113	PIR1.2 [41]; PIR2 [41]
	HAI3	PIR1.2 [41]; PIR2 [41]
SnRK2 kinases	SnRK2.3/SRK2I	PP2-B11 [42]
	SnRK2.6/OST1	HOS15 [43]
ABA-responsive transcription factors	ABI5	DWA1 [44]; DWA2 [44]; KEG [45]; ABD1 [46]; COP9 [47]
	ABI3	AIP2 [48,49]
	ABI4	COP1 [50]
	ABF1	KEG [51,52]
	ABF2/AREB1	KEG [53]
	ABF3/AREB2	KEG [51,52]
	DREB2	DRIP1 [54]; DRIP2 [54]; BPM2 [55]
	HB6	BTB1-6/BPM1-6 [56]
	MYB96	MIEL1 [57] (Myb96 activates ABI4)
	MYB30	RHA2b [58]; MIEL1 [57,59]
	RAV1	BPM1 [60,61]
Other regulatory and signaling factors and ABA-responding genes	ADA2B	SKIP24/At1g08710 [62]
	ATP1/SDIRIP1	AIRIP2 [63,64]; AIRP1 [63,64]; SDIR1 [65]; (SDIRIP1 activates ABI5)
	ACD11	XBAT35.2 [66]; (ACD11 is one of ABA-responding genes)
	RD21	AIRP3/LOG2 [67]; (positive role in ABA responses; RD21 is Cys protease)
	CIPK26	KEG [68,69]
	PP2A	CHIP [70]

**Table 2:** E3 ligases with unidentified targets that are known to regulate ABA responses. The original references are provided next to the E3 ligase; however, this table is a compilation of the tables from several earlier reviews [6,7,12,99,100], as well as new data that were not previously reviewed.

E3 Family	E3 Ligase [Reference]	Influence on ABA Response/Remarks
RING-type	ATL61 [101]	Positive regulator in the ABA-mediated drought stress response
SCF	ARKP1 [102]	Positive role in ABA signaling network; mutants displaying reduced ABA-mediated inhibition of seed germination, root elongation, and water loss rate of detached leaves
RING-type	CHYR1/RZP34 [103]	Promotes ABA-induced stomatal closure, reactive oxygen species production, and plant drought tolerance; activity is regulated by SnRK2.6
RING-type	SINA2 [104]	Regulates plant responses to ABA and osmotic stress; activity is regulated by CDKG1 (cyclin-dependent kinase G1)
RING-type	AtPPRT1 [105,106]	Negative role in ABA and drought stress responses
RING-type	AtARRE/ATL27 [107]	Negative regulation in ABA signaling
BTB-type	BPH1 [108]	Negatively involved in ABA-mediated cellular events; mutation caused delayed seed germination in response to ABA and resulted in hyper-induction of a large portion of ABA-inducible genes in response to ABA; mutants exhibited enhanced stomatal closure under ABA application and reduced water loss
RING-type	JUL1 [109]	ABA-mediated microtubule disorganization; regulates stomatal closure, and tolerance to drought stress
RING-type	COP1 [110]	ABA-mediated microtubule disorganization, stomatal closure
RING-type	RDUF1/2 [111]	Negatively regulates ABA signaling
RING-type	RHA2a/2b [112,113]	Positively regulates ABA signaling
RING-type	AIRP4 [114]	Positively regulates ABA signaling
SCF	TLP3/9 [115]	Positively regulates ABA signaling
RING-type	ATL43 [116]	Positively regulates ABA signaling
RING-type	CER9 [117]	Negatively regulates ABA signaling
U-box	PUB9 [118]	Negatively regulates ABA signaling
U-box	PUB18/19 [119,120]	Negatively regulates plant drought response and ABA signaling
SCF	DOR [121]	Negatively regulates ABA signaling
SCF	EDL3 [122]	Positive regulator in seed germination and root growth; positively regulates ABA signaling
SCF	MAX2 [123]	Negatively regulates plant drought stress response through mediating ABA signaling; negatively regulates ABA signaling
DDB	DWA3 [124]	Negatively regulates ABA signaling; mutants exhibited ABA-hypersensitivity
U-box	PUB44/SAUL1 [125]	Negatively regulates ABA signaling
RING-type	XERICO [126]	Positively regulates ABA-dependent drought response; overexpression leads to ABA over-accumulation
SCF	FOF2 [127]	Plays an important negative role in ABA-mediated seed germination and early seedling development, as well as a positive role in ABA-mediated drought tolerance
